# Supplementary material for: Complexome profiling of the Chlamydomonas psb28 mutant reveals TEF5 as an early PSII assembly factor
Source: Plant Cell. 2025 Mar 17;37(6):koaf055. doi: 10.1093/plcell/koaf055 (PMC12164590; doi:10.1093/plcell/koaf055)
Supplement: koaf055_Supplementary_Data [file koaf055_supplementary_data.zip › psb28_interactive.zip!/plots/intensity/qualityPlots/peptides/CTP4_Cre10.g422201.t1.1.html]

### Cre10.g422201.t1.1

Trivial name: CTP4  
SeqCoverage [median]: 6.20  
Mass: 102.09 kDa  
  
 

| Sequence | MissedCleavage | Mass [Da] | wtIntensity | muIntensity | Score |
| --- | --- | --- | --- | --- | --- |
| ATATGAASTVAGIAR | 0 | 1316.70 | 0.00 | 372.55 | 76.65 |
| DTLRPDAAATVAALK | 1 | 1511.83 | 0.00 | 492.90 | 76.76 |
| LAAFISQR | 0 | 904.51 | 0.00 | 257.52 | 67.03 |
| LAAVDTVVLDKTGTLTEGK | 1 | 1930.06 | 0.00 | 611.13 | 41.87 |
| LVSDAQAR | 0 | 858.46 | 0.00 | 384.92 | 68.92 |
|  |  |  | 0.0 | 2119.0 |  |
